# Collaborative agroforestry to mitigate wildfires in Extremadura, Spain: land manager motivations and perceptions of outcomes, benefits, and policy needs

**DOI:** 10.1007/s10457-022-00771-6

**Published:** 2022-10-10

**Authors:** Franziska Wolpert, Cristina Quintas-Soriano, Fernando Pulido, Lynn Huntsinger, Tobias Plieninger

**Affiliations:** 1 Faculty of Organic Agricultural Sciences, University of Kassel, Steinstraße 19, 37213 Witzenhausen, Germany; 2 Biology and Geology Department, Andalusian Center for the Assessment and Monitoring of Global Change (CAESCG), University of Almeria, Almería, Spain; 3 Institute for Dehesa Research (INDEHESA), University of Extremadura, Plasencia, Spain; 4 Department of Environmental Science, Policy, and Management (ESPM), University of California, Berkeley, USA; 5 Department of Agricultural Economics and Rural Development, University of Göttingen, 37073 Göttingen, Germany

**Keywords:** Productive fuelbreaks, Wildfire mitigation, Mediterranean, Silvopastoralism, Agroforestry, Land abandonment, Integrated landscape management

## Abstract

Wildfires are increasing in severity, and magnitude in the Mediterranean Basin in recent years, reaching a yearly average of 450 000 ha over the last decade. Drivers include climate change, land-use change, and land abandonment. Wildfire mitigation requires landscape-level action as impact to each parcel is affected by the conditions of the others. We conducted a case study of a regional-level initiative that develops community efforts to mitigate wildfires through silvo-pastoral agroforestry systems, using an *integrated landscape management* approach. This approach involves collaboration among stakeholders to achieve multiple objectives. In order to derive insights into its potential, we asked participating land managers: (1) What motivates their participation?, (2) How do they perceive initiative outcomes?, and as urban outmigrants with non-traditional goals are increasing in rural areas, (3) Do responses differ between rural and neo-rural participants? Our results show that managers feel highly affected by wildfires and are strongly motivated to reduce wildfire risk. Land abandonment and inappropriate policy were major concerns. The initiative was seen to have positive outcomes for individual participants as well as the region, and to stimulate community connectedness. We conclude that fit to local contexts, integrated landscape management can be a well-received approach to reducing wildfire risk. Agroforestry systems in Extremadura can act as “productive fuelbreaks” that reduce fire risk over extensive areas, while restoring traditional landscapes. We suggest that programs to reduce wildfire risk can also be used as a leverage point for financing rural revival and provision of multiple ecosystem services.

**Supplementary Information:**

The online version contains supplementary material available at 10.1007/s10457-022-00771-6.

## Introduction

Mediterranean vegetation—a mosaic of shrublands, woodlands, pastures, and fields—is wildfire prone. Mild and wet winters promote biomass accumulation and are followed by hot summers that make the vegetation dry and flammable (Keeley et al. 2012; Moreira et al. 2020). Historically, intentional, low intensity burning was a common land management practice based on traditional know-how, and used to expand pasture and cropland (Rego et al. 2010). Clearing dense vegetation contributed to a diverse landscape and reduced fuel loads (Ortega et al. 2012; Damianidis et al. 2021). However, things have changed. Today, one of the major causes of wildfires is escaped fire from intentional burning (Rego et al. 2010). In recent years, hot and fast spreading fires, so called megafires, increasingly threaten whole social-ecological systems and have become a problem for Mediterranean regions globally (Lindenmayer and Taylor 2020; Safford et al. 2022). In the last decade an annual average of 450 000 ha have been burned in the Mediterranean Basin (FAO and Plan Bleu 2018). Large fires are defined as fires that affect more than 500 ha and cannot be controlled due to flame size, fire speed, or canopy fire (Alló and Loureiro 2020). Drivers include climate change, land-use change, land abandonment and short-sighted fire suppression policies (Moreira et al. 2011, 2020; Moreno et al. 2014; Gan et al. 2015; Varela et al. 2020).

Mediterranean rural landscapes are subject to land abandonment and rural depopulation (Azevedo et al. 2011). The resulting land use change challenges the biodiversity and ecosystem services supported by traditional agro-silvo-pastoral systems characteristic of these areas (Varela et al. 2020; Quintas-Soriano et al. 2022). Without grazing, burning, cultivation, or clearing to keep regrowth in check, abandoned lands and burned areas become dense shrublands and forests, increasing fuel loads, and creating continuous fuels fostering wildfire spread (Varela et al. 2020). Such lack of forest management results in larger, hotter, and faster spreading wildfires (Damianidis et al. 2021).

For decades, existing top-down wildfire mitigation policies have focused on fire suppression in Spain and other Mediterranean regions (Moreira et al. 2020). However, the result is a “fire paradox”: when fires are suppressed, absent other vegetation control methods, vegetation grows freely, and biomass accumulations build fuel loads over time, eventually feeding megafires (Rego et al. 2010). Creating fire-resistant landscapes (DeRose and Long 2014) has therefore emerged as key to reducing large wildfires (Moreira et al. 2020). One option is creating a network of linear strips of bare soil (fire breaks) or low biomass vegetation (fuel breaks) (Ascoli et al. 2018). Fire and fuel breaks can slow down fire spread and can act as an anchor for fire suppression (Duguy et al. 2007; Oliveira et al. 2016). However it is necessary to transform a high percentage of the landscape (e.g. 20–30%) into fuel or fire breaks to effectively change fire incidence (Oliveira et al. 2016), calling for the integration of local community engagement into wildfire mitigation at the landscape scale. Payment schemes for implementing fire breaks and fuels reduction through shrub clearing and/or grazing have been successfully implemented, for example, in La Rioja and Andalusia (Lasanta et al. 2018; Varela et al. 2018).

Implementing and maintaining agroforestry systems can be an important pathway for mitigation wildfire risk by decreasing fuel loads, changing fuel characteristics, and acting as fuel breaks that cover extensive areas (Moreira et al. 2020; Damianidis et al. 2021). They can maintain aesthetically pleasing landscapes, provide products for human use, and support carbon sequestration in trees unlikely to be consumed by fire. Trees are fewer than in forests and spaced more widely, while management for grazing and/or cropping results in less continuous understory biomass and less woody vegetation than in unmanaged grasslands and shrublands (Varela et al. 2020; Damianidis et al. 2021). In the Spanish region of Extremadura, they may also restore and maintain traditional agro-silvo-pastoral landscapes such as *dehesa*. Dehesa landscapes have been found to be among the most fire-resistant in Spain but are in decline, while more fire-prone landscapes have increased (Ortega et al. 2012). Extensive agroforestry systems can act as “productive fuelbreaks” for communities surrounded by fire-prone vegetation (Bertomeu et al. 2022).

Essential components of successful wildfire mitigation are bottom-up strategies with region-wide stakeholder collaboration (Gan et al. 2015). Worldwide, such multi-stakeholder collaborations have been promoted under the umbrella of “integrated landscape initiatives.” An integrated landscape initiative is a group of people from different sectors with common goals, supporting a variety of landscape values. They actively engage in land management, awareness raising, and education (García-Martín et al. 2016; Carmenta et al. 2020). In many parts of Europe, *neo-rurals* (people that have moved in the last two decades from urban to rural areas for living and working on the land) play a role in integrated landscape management as they are growing in number and often seek new models of sustainable land management, the experience of living close to nature, and engagement in local, healthy food production (Escribano and Mormont 2007; Orria and Luise 2007).

Considered a holistic approach to landscape management (García-Martín et al. 2016), integrated landscape initiatives are increasingly supported by funding bodies at local to global scales (Sayer et al. 2017). In recent years, “landscape thinking” and the need to empower rural communities has been widely recognized in risk mitigation strategies, and in particular as a complement to top-down wildfire suppression approaches (Prior and Eriksen 2013; Carroll and Paveglio 2016). Collective engagement in wildfire mitigation in the Mediterranean Basin has been analysed by Górriz-Mifsud et al. (2019), with a focus on community-based fire preparedness and suppression. How to expand fuel treatment strategies to the landscape scale on Lesvos island, Greece, was studied by Palaiologou et al. (2020). Otero et al. (2018) did research on integrating local communities into decision making for wildfire suppression and preventive mitigation planning in Catalonia, Spain. However, little is currently known about participant motivations and perceptions of the outcomes of integrated landscape initiatives in wildfire mitigation. In particular, the role of stakeholder cooperation in land management in relation to the use of traditional practices and local knowledge has not yet been studied. Here, we contribute to the literature the perspectives of diverse land managers on wildfire mitigation. Our study aims to explore the social-ecological dimensions of the integrated landscape initiative in Extremadura, Spain, known as “MOSAICO” (further referred to as “the initiative”). The Initiative seeks to reduce the impact of wildfires through management of fire-resistant multifunctional mosaic landscapes and use of productive fuel breaks that are often adaptations of traditional agricultural systems, most notably silvo-pastoral agroforestry. Drawing on a survey of participating land managers, we address the following questions: (1) What motivates land manager participation?, (2) How do participants perceive the outcomes of the integrated landscape initiative? And, (3) Are there differences in responses about motivations, barriers, outcomes, and wildfire-related measures between rural and neo-rural land managers? We present our results and discuss the integrated landscape initiative as a model for collaborative wildfire mitigation, highlighting agroforestry as a tool for promoting fire-resistant landscapes, and closing with policy recommendations.

## Methods

We chose an in-depth case study approach aiming for holistic insights in a complex field (Brown 2008). The approach allows in-depth, multi-faceted explorations of complex issues in their real-life settings. It provides the opportunity to explore the key characteristics, meanings, and implications of the topic, identifying areas for further research (Crowe et al. 2011).

### Study area and local context

**Fig. 1:**
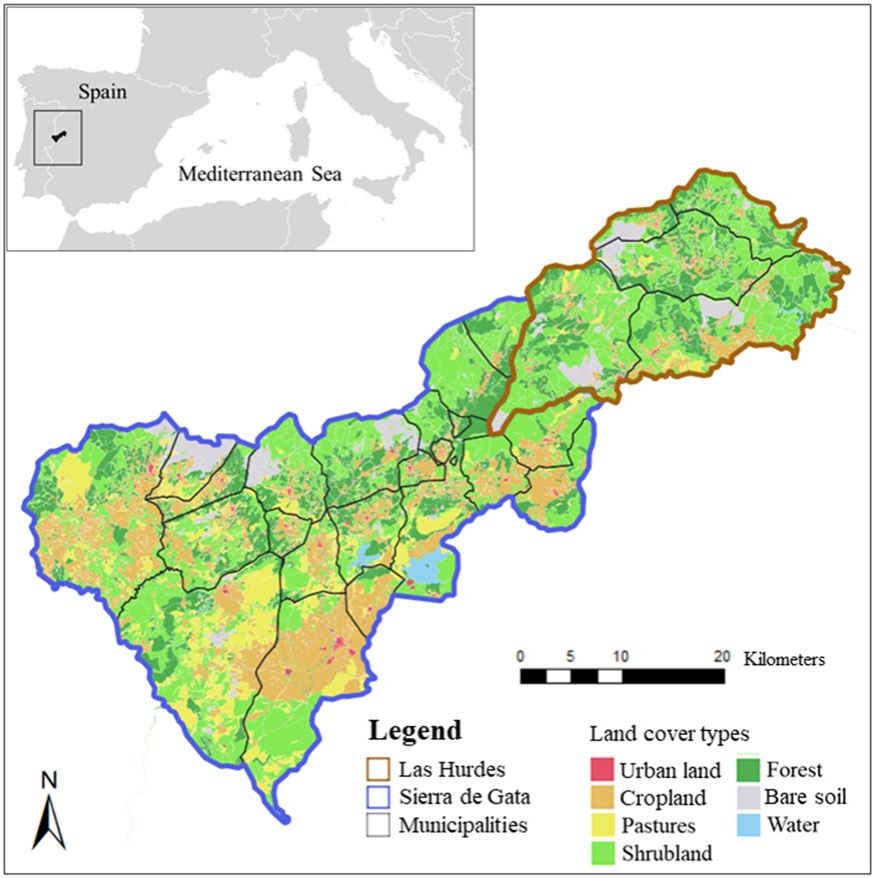
Maps of the study site, the counties of Gata and Las Hurdes in Extremadura, Spain (REDIAM 2007)

The case study area is in a rural part of western Spain, the adjacent counties of Sierra de Gata and Las Hurdes in northern Cáceres Province of the Extremadura Autonomous Region (Fig. 1). Sierra de Gata is 1257.94 km^2^ in size with 19 municipalities. The initiative is active in several of these municipalities such as the municipality of Valverde del Fresno with 2250 inhabitants and Gata with 1413 inhabitants. Las Hurdes is 499.37 km^2^ in size and consists of 6 municipalities, including the largest two, Caminomorisco with 1181 inhabitants, and Pinofranqueado with 1692 inhabitants (IEEX 2021). The climate in the area is typically Mediterranean, with mild, wet winters and hot, dry summers.

Sierra de Gata and Las Hurdes are far away from major transportation routes. Isolation has contributed to local development of a rich cultural heritage and ecological knowledge linked to traditional landscape management (Catani 2004; Solymosi 2011). The landscape was largely a mosaic of agroforestry uses, dominated by pasture with tree crops (Montiel-Molina et al. 2019). Dry stone terraces used for fruit and vegetable cultivation have been common (Abel-Schaad et al. 2014). The afforestation policy of the Franco regime (1940–1975) resulted in massive pine plantations which are positively correlated with forest fire occurrence (Iriarte-Goñi and Ayuda 2018). Since the 1950s, industrialisation and socio-economic crises have fueled outmigration, leaving a population of rising average age (Madruga et al. 2021). This rural depopulation also caused land abandonment, abandonment of livestock grazing and resulting in forest encroachment, and in consequence flammable biomass accumulation and a more fires (Iriarte-Goñi and Ayuda 2018). The traditional agroforestry that once blanketed the rough topography of our study region has substantially decreased in area, first in Las Hurdes (since the 1930s) and later in Sierra de Gata (since the 1960s). Nowadays, national and regional regulations hamper land use change from forest to agricultural land, and grazing is rarely allowed in public forests. An abandoned agroforestry system crowded with trees is typically reclassified as forest, limiting its use for livestock husbandry and cultivation. If a forest burns down, the land can be converted to farmland only after 30 years. Forest ownership is related to forest condition, with public forests receiving the highest investment in silvicultural treatments and fire suppression infrastructure. Private forests are short of active management due to low or no profitability, except in those areas managed under public–private agreements. In Sierra the Gata and Las Hurdes, 2.298 wildfires burned on 37.500 ha between 2000 and 2015 (Bertomeu et al. 2019). Despite a decline in fire occurrence and burned area between 1983 and 2021, a greater fraction of area was burned in large (> 500 ha) or very large (> 5000 ha) fires (Ministerio de Transición Ecológica 2022). In 2015, a single megafire in Sierra de Gata burned nearly 8000 ha (Bertomeu et al. 2022). In the region, most resources are allocated to fire suppression infrastructure (most commonly firebreaks and firefighting equipment). Prevention is generally small-scale fuel removal treatments around cities and preventive silvicultural treatments in pine stands.

### The MOSAICO initiative

The major aim of the MOSAICO initiative in Sierra de Gata and Las Hurdes is to foster mutual learning among local stakeholders and collaboratively engage in wildfire mitigation using “productive fuel breaks,” areas maintained by agroforestry practices (Varela et al. 2020). The initiative is supported by the University of Extremadura, the Government of Extremadura, and the European Union. Land managers apply to for initiative membership, and are accepted if they contribute to fuel reduction through forest management, livestock grazing, crop cultivation, or agroforestry. Examples of such activities include establishment of goat herding, planting of fruit trees, resin harvesting, pine tree biomass harvesting, and implementation of new practices like rotational grazing. The average size of properties managed as part of the initiative is 63.8 ha. The initiative provides administrative, field technical advice, and other services, including support in completing and submitting funding applications.

### Survey design

Our questionnaire sought insight into land manager perceptions of the integrated landscape initiative and consisted of 7 thematic sections about: (1) land managers characteristics, (2) land managers activities (3) aims/motivation, (4) perceived outcomes/performance of the initiative, (5) perceived barriers to management success, (6) perceived success factors for initiative goals, and (7) perceptions of wildfires (Supplementary Material 1). We developed questions and statements covering these themes after intense discussions with experts in the region. Most answer options were in a likert scale format, i.e. for each the respondents had to indicate their level of agreement on a scale from 1 to 5 (e.g. 1 = strongly disagree to 5 = strongly agree, with 3 indicating neither agree or disagree) (Joshi et al. 2015). In some cases, respondents could complement predefined answers with their own options (e.g. motivations). To help explain and supplement answers to predefined questions, and to allow respondents to add issues they felt were missing in the predefined questions, we added open-ended questions (e.g. on outcomes of the initiative).

### Data collection and analysis

We surveyed land managers that were part of the integrated landscape initiative MOSAICO (Varela et al. 2020; Bertomeu et al. 2022). Some landowners may not live on or manage the land. We are interested in the land managers perceptions, who are actively involved in full or part time land management and often live on the land. Contact information for 141 land managers was provided by the initiative. We aimed to include all land managers that considered themselves active members. Applying this criterion reduced eligible respondents to 95. Out of these 95, 10 declined participation and 19 were not available via phone and/or did not respond to our emails. In the end we conducted 66 interviews, corresponding to a rather high response rate of 69% (García-Martín et al. 2016; Carmenta et al. 2020). Wherever possible, face-to-face interviews were conducted by field assistants from September to December 2020. Enumerators followed safety protocols for COVID-19 risk. Informed consent was obtained.

Nine respondents prefered telephone, two e-mail interviews. Field assistants recorded participant answers for digitizing and translating into English. Of the 66 respondents, three responses had to be removed from the analysis because interviews revealed that they were not actively engaged in land management, so a total of 63 surveys were used for the analysis.

Due to the exploratory character of our study (and as variance of responses was low across all categories), we most often used frequency analysis. We calculated response mean values and ranked them according to levels of agreement. For the comparison of rural versus neo-rural participants, we conducted nonparametric statistical comparison analysis (Mann Whitney test) including 62 surveys, as one respondent could not be identified as rural or neo-rural. Answers to open-ended questions were used to support, supplement or challenge the findings of the quantitative analysis.

## Results

### Land managers and farming activities

The majority of land managers were 36 to 50 years old (57%). 14% were younger, 24% were 51 to 65 and a very few (5%) were 65 + years old. Of the interviewed land managers 27% were female. With 42%, nearly half were neo-rurals. Participation in the initiative lasted from 1 to 5 years and a similar number of people joined the initiative each year leading to our cumulative total participants. The majority of respondents practiced land management as a side job–62% earned 25% or less of household income from farming activities. Only 19% of farming activities contributed 76–100% to household income, while 11% of land managers earned 51–75% and 8% of land managers earned 26–50% of household income from land management. Farms were mostly managed by single persons (38%) or families (44%), only 10% of the farms had 2–5 workers and 8% had more than 5 workers.

**Fig. 2:**
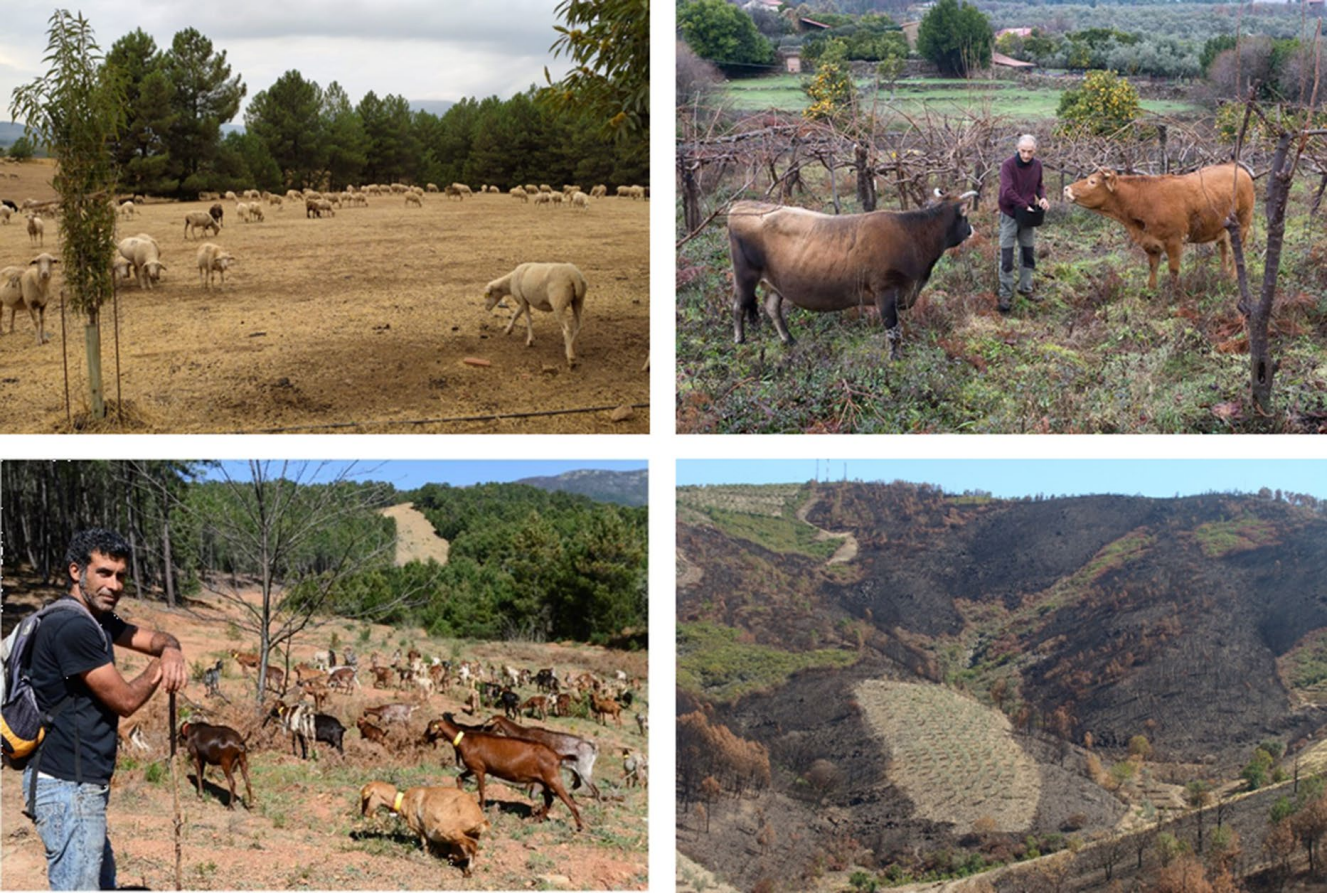
Common agroforestry practices in the integrated landscape initiative: Sheep and sweet chestnuts (top left), cows with kiwi (top right), goat herding in a semi-open landscape (bottom left), unburned grazed fruit orchard surrounded by burned forests (bottom right). Note the discontinuous tree canopies and the sparce understory fuels in the agroforestry systems

Land managers had between one and ten different activities on their farm (Tab. 1). Farms were often agroforestry systems, e.g. sheep husbandry in a chestnut orchard (Fig. 2). The most common land management activities were olive and chestnut orchards, livestock husbandry, and agroforestry. Other fruit trees grown included cherries, almonds, pistachios and figs. Around 15% of land managers produced fuelwood, resin, timber, dairy and/or aromatic plants. Production of honey, vegetables, herbs, poultry, cereals, cork and snails as farming activities was rare. In an open-ended question, we asked the respondents how they defined themselves as a land manager. We got diverse answers such as: “a farmer for hobby and entertainment; as a motivated beginner; as a caretaker, responsible for the environment; as a happy farmer; as a rural farmer and rancher; as an example for people to follow; as a fighter for agroforestry.”

**Table 1:** Respondent’s most common activities on their farms

Within-farm activities	Portion of all farms [%]	Within-farm activities	Portion of all farms [%]
Livestock husbandry	40	Wood fuel	16
Olive trees	35	Resin tapping	14
Agroforestry	32	Forestry for wood	14
Chestnut trees	32	Dairy farming	14
Other fruit trees	27	Aromatic plants	14

### Wildfires: Impacts and approaches

**Fig. 3:**
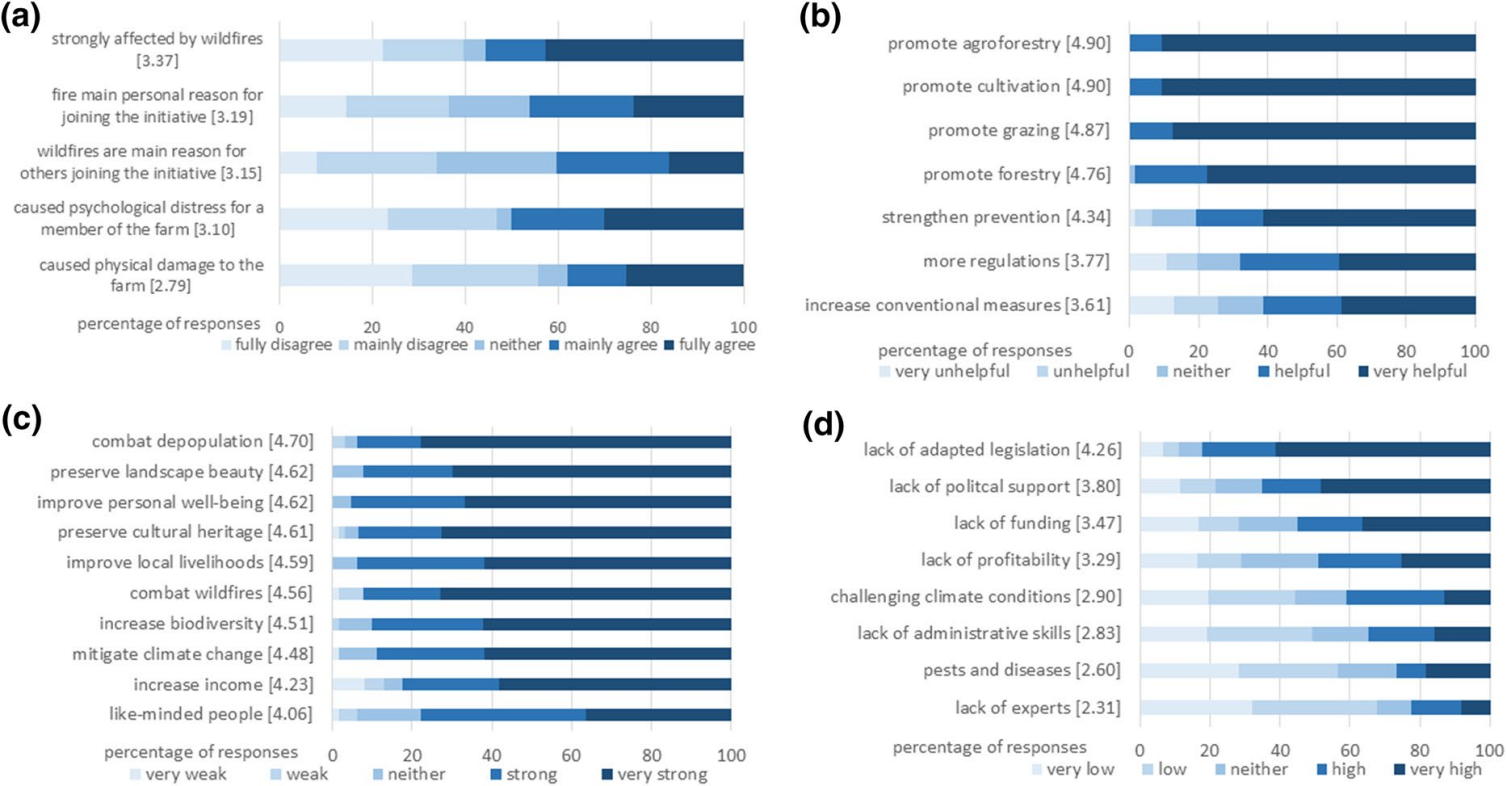
Farmer perceptions of **a** Wildfire impacts, **b** Measures against wildfires, **c** Motivations for land management and **d** Barriers to success. Color intensity reflect answer categories. Mean values are shown in brackets. (Color figure online)

Respondents were asked about wildfire impacts and suitable measures for combating them. More than half fully or mainly agreed they were strongly affected by wildfire (Fig. 3a), with the vast majority in full agreement. Only a fifth fully disagreed that they were strongly affected. Half mainly or fully agreed that wildfires caused psychological distress for a member of their farm. Nearly 40% of the farms were physically damaged by wildfire. About half of the land managers fully or mainly agreed that combating wildfire was their main reason for joining the initiative.

We listed potential measures against wildfires and asked about their usefulness (Fig. 3b). All land managers indicated that three were either very helpful or helpful: “promoting agroforestry,” “promoting cultivation” and “promoting grazing.” Almost all managers agreed with “promoting forestry.” There was some disagreement with “strengthening prevention,” “more regulations,” and “increasing resources for conventional measures,” such as increasing number of fire-fighting helicopters, though more than half still agreed these were helpful or very helpful.

### Motivations

We asked about the importance of various motivations for their land management as part of the initiative (Fig. 3c). Over 80% agreed that most of the items listed strongly or very strongly motivated them to engage in land management and the initiative. The most motivating was “combating depopulation” followed by “preserving landscape beauty” and “improving personal well-being,” with no respondents ranking them as weak or very weak motivations. These were followed by “preserving cultural heritage,” “improving local livelihoods,” and “combating wildfires.”

### Barriers to success

We asked land managers to agree or disagree with statements about the severity of possible barriers to success for their activities (Fig. 3d). Interestingly, lack of legislation adapted to the current fire situation and of political support were perceived as having a greater negative impact than a lack of funding and profitability. More than half of the land managers found a “lack of adapted legislation” to be a high or very high barrier. The barrier with the second highest impact was a “lack of political support,” followed by a “lack of funding” and a “lack of profitability.” Lack of experts was considered the lowest barrier.

### Outcomes

**Fig. 4:**
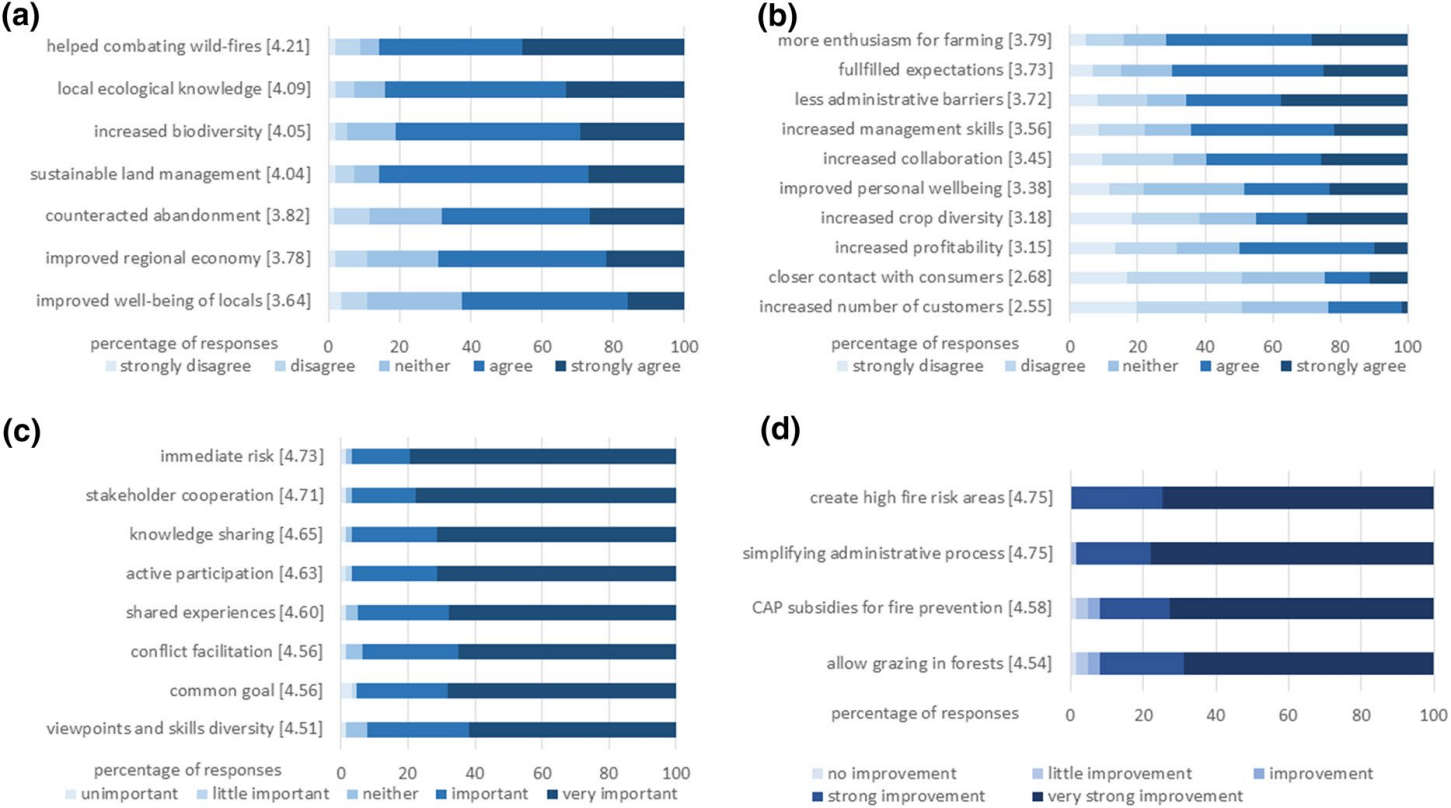
Farmer perceptions of **a** Regional outcomes, **b** Personal outcomes, **c** Success factors and **d** Policy recommendations. Color intensity reflects answer categories. Mean values are shown in brackets. (Color figure online)

We enquired about perceived regional and personal outcomes of the initiative. Regarding regional outcomes, “helped combating wildfires” was agreed with by the most respondents (Fig. 4a). “Increased local ecological knowledge” was second, very closely followed by “increased biodiversity” and “increased sustainable land management.” Over 80% agreed or strongly agreed with four statements above, and only 2% strongly disagreed. “Counteracted abandonment,” “improved the regional economy,” and “improved the well-being of locals” were agreed with by more than half of the land managers, while only 2 to 4% strongly disagreed with them.

The rate of agreement about personal outcomes was more differentiated (Fig. 4b). The strongest agreement, by more than half, was that they had “more enthusiasm about traditional land management.” There was a similar distribution of agreement with “fullfilled personal expectations.” As we found out from an open-ended question, this fulfillment mostly referred to advice and consultation for land management and coping with bureaucracy (about 30% of participants). About 13% of respondents stated in the open-ended question that collaboration, combating wildfires, or an increase in yield/profitability from joining the initiative were expections fulfilled. There were also a few that mentioned that fighting abandonment fulfilled personal expectations.

The third personal outcome most respondents agreed with, and with the highest percentage strongly agreeing, was that the initiative “helped to overcome administrative barriers.” This was followed by “increased management skills,” “increased collaboration among land managers,” “improved personal wellbeing,” “increased crop diversity,” “increased profitability,” and “led to closer contact with consumers.” The last, “increased customers,” still had a fourth of respondents agreeing with it, though few strongly agreed.

To complement the statements about outcomes, we asked the land managers in an open question about what had changed on their farm since they joined the initiative. About half of stated that there were no changes. Some further explained that they are still in the initial stages of the common project so it was too early to say. The most frequent change was gaining knowledge and advice. Changing crops, cultivation of abandoned land, and changing grazing methods to rotational grazing to avoid overgrazing were mentioned 4 times. More focus on fruit trees was mentioned 3 times, especially chestnuts (mentioned twice). Other outcomes mentioned were changing farming techniques, starting to label products, and no longer feeling alone. One stated that he now sees “the natural environment from another perspective.”

### Success factors

We asked respondents to agree or disagree with the importance of possible success factors for the initiative. The majority (over 60%) of land managers perceived all the suggested success factors as very important (Fig. 4c). The most important was “fighting a common and immediate risk like fire.” Second was “cooperation between different stakeholders and sectors.” This was followed by “knowledge sharing,” “active participation,” “shared experiences,” “conflict facilitation,” “having a common goal,” and having “a diversity of viewpoints and skills.”

### Policy support

We asked respondents to assess policy options in terms how important each would be for improving wildfire mitigation. All options received strong support from land managers (Fig. 4d). All agreed that creating a special land management regime would be an improvement. Simplifying the administrative process was considered a very important possible improvement by most, for another fifth it was a strong improvement and very few agreed with little improvement. This high agreement also shows that MOSAICO administrative advice is important to participants, including help for establishing an enterprise, applying for CAP subsidies, and requesting permission for special land management (like cutting or planting trees). “Make changes in the Common Agricultural Policy (CAP) to subsidize the fire mitigation service provided by land managers” was regarded as a very strong or strong potential improvement by almost all. The lowest ranked of the four policy support options, “Allow grazing in forests” was still perceived as potentially a very strong improvement by more than two-thirds of the land managers and as “a strong improvement” by another fourth.

### Differences across rurals versus neo-rurals

Neo-rural and rural land managers differed in some of their responses, especially for motivations and wildfire measures (see Suplementary Material 2). Neo-rural respondents showed higher motivations compared to rurals on: increasing biodiversity (*U* = 387.5; *p* = 0.012), growing their own food (*U* = 273.5; *p* = 0.003) and improving personal wellbeing (*U* = 323.5; *p* = 0.010), while rurals were more motivated by mitigating climate change compared to neo-rurals (*U* = 409.5; *p* = 0.019). Neo-rurals perceived pest and diseases as a higher barrier than rurals (*U* = 320.0; *p* = 0.029). We did not find statistical differences among further perceived barriers and outcomes. Regarding measures to mitigate wildfires, rurals rather than neo-rurals more often perceived the promotion of grazing (*U* = 488.0; *p* < 0.0001) and cultivation (*U* = 483.0; *p* < 0.0001) as helpful, while neo-rurals were more favorably inclined toward agroforestry (*U* = 452.0; *p* < 0.0001) as a helpful measure, although both groups mainly agreed to the helpfullness of all three measures.

## Discussion

Large-scale catastropic wildfires are on the rise in the Mediterranean region, and there is increasing awareness that preventing and reducing their impacts most often requires cooperation among land managers at the landscape level. To understand the complexities of such cooperation, we performed a first exploratory survey of a community-based initiative for wildfire mitigation in Europe, providing insights into land manager perceptions of their motivations for participation, and of initiative barriers to success and outcomes for the individual as well as for the local population. Land managers found collaborative wildfire management was multifunctional, reducing fire hazard, reviving abandoned landscapes, and increasing biodiversity. Here we discuss how the investigated initiative offers a model for collaborative action with multiple benefits, highlighting the role of agroforestry, and then close with policy recommendations and conclusions.

### A model for collaborative wildfire mitigation

The highest level of agreement about regional outcomes was that integrated landscape management “helped in combating wildfires,” meeting the initiatives’ main objective and making it a success for its members. We want to stress that our study is based on the perceptions of respondents, and these can be influenced by contextual factors notably including participation in social networks. The realised impact of the initiative regarding fire risk and potential spread is analysed in Bertomeu et al. (2022).

Reduction of fire risk was a main driver for collaborative action, and previous research has found that reducing fire risk is a common motivation for California landowner cooperation as reported by landowners (Ferranto et al. 2013). Our respondents agreed that wildfire impacts were broad and multifaceted, including causing psychological distress that touched land managers in half of the studied farms. This is an impact that has been somewhat neglected in the literature (Finlay et al. 2012; Waks et al. 2019).

Typically, integrated landscape initiatives develop to attempt to resolve land use conflicts, for example such as the spread of extractive industries into cultural landscapes, or when biodiversity conservation creates tradeoffs with livelihoods (Sayer et al. 2015). In contrast, our studied initiative seeks to collaboratively reduce wildfire risk by reviving management of abandoned land (Bertomeu et al. 2022). This is a new and globally important domain where integrated landscape initiatives can take meaningful action. Social cohesion is a key factor in creating a wildfire resistant and resilient community because wildfire risk reduction cannot be tackled effectively by individuals (Prior and Eriksen 2013; Townshend et al. 2015). Prior and Eriksen (2013) found in particular that community characteristics like “sense of community” and “collective problem solving” support adoption of fire preparation practices and the development of cognitive capacities that reduce vulnerability and support collaborative action. We found increased collaboration to be an outcome highlighted by respondents, an indicator of social cohesion. The shared immediate risk of wildfires, and the experience of developing and carrying out initiatives to reduce wildfire, pushed land managers to develop common purpose and shared goals. In our case, integrated landscape management promoted social cohesion via a framework for community wildfire mitigation. Similarly, Prior and Eriksen (2013) point out that community efforts should be acknowledged for their role in shaping the beliefs and attitudes of the participants. Effective development of shared goals and practices calls for engagement of people in risk communication and mitigation activities, rather than passive transfers of information (Tedim et al. 2016). Taking action ultimately relies on individual beliefs about what is meaningful, important and possible. Focus on individual and community empowerment can prevent being overwhelmed by a global-scale problem (Prior and Eriksen 2013). Local to regional efforts in collaborative action to solve environmental problems are at a level that empowers local people to actively engage and gives a feeling of self-efficacy (Górriz-Mifsud et al. 2019).

### Agroforestry for fire resistant landscapes

In addition to reducing fire risk, establishing agroforestry systems has a critical role in sustainable and regenerative land management globally (Plieninger et al. 2020; Damianidis et al. 2021). Perceived increases in biodiversity and human wellbeing have often been achieved through the expansion of agroforestry systems (Damianidis et al. 2021). For instance, in an abandoned landscape, agroforestry practices help enhance diversity by restoring openings in the canopy and increasing habitat diversity (Varela et al. 2020). They also enhance carbon sequestration by retaining trees (Kay et al. 2019) and reducing the likelihood of fire risk (Damianidis et al. 2021).

Moreira et al. (2011) identified three strategies for fire resistant landscapes: creating and maintaining productive landscape-scale fuel breaks, reducing fuel loads, and substituting fire-prone species with more fire-resistant ones. Agroforestry systems, such the multitude of fruit orchards that form part of MOSAICO, encompass all these strategies: they reduce fire risk by establishing and maintaining productive fuel breaks, shrublands or pine forests are replaced with less fire prone vegetation and vegetation structure (e.g. chestnut orchards with sheep), and grazing reduces understory fuels and suppresses woody vegetation. Before land use abandonment, Sierra de Gata and Las Hurdes were models for fire resistant tree crop systems managed with grazing and forest clearing (Montiel-Molina et al. 2019).

When fire damage to agricultural and forestry goods is accounted for, Spanish silvopastoral agroforestry systems are more profitable than timber production alone (Moreno et al. 2014). Restoring burnt areas between 2013 and 2017 in Spain cost almost 70 million Euros. Spain is the country with the highest vulnerability to land degradation among European countries (Varela et al. 2020). To tackle these problems, the Catalonian Government has released a “Forest Policy General Plan” that suggests different management tools for decreasing fire risk. Casals et al. (2009) emphazise the importance of agroforestry to the Catalonian government’s fire prevention plan. Animal grazing not only reduces wildfire risk and conserves biodiversity, but it is relatively inexpensive, offering a viable alternative to increasingly costly yet failing conventional supression measures (Bertomeu et al. 2022). Especially in combination with shrub clearing, livestock grazing is a effective tool in wildfire risk reduction (Lasanta et al. 2018). Animal grazing can also complement prescribed burning, reducing the hazard of escape with lower fuel loads (Rigolot et al. 2009; Davies et al. 2016).

Our respondents’ perceptions were confirmed by a review on land cover and wildfire relations that identified grasslands and farmland as options for decreasing wildfire vulnerability (Moreira et al. 2011). Data from the northern Mediterranean reveals that agroforestry systems are less affected by wildfire, compared to forests, shrublands, or grasslands, and are also environmentally friendly and contribute to human well-being (Carmo et al. 2011; Damianidis et al. 2021). Strong agreement that forest harvest and management were very helpful for wildfire mitigation concurs with the high fire risk found in abandoned forests (Azevedo et al. 2011; Badia et al. 2019; Montiel-Molina et al. 2019).

### Revival of rural cultural landscapes

Sierra de Gata and Las Hurdes are cultural landscape hotspots for their unique but threatened terraced landscapes. From 1960 to 1975, Extremadura lost about one third of its inhabitants due to emigration to cities–in some counties half of the people left, leaving an aging society behind (Rosado 2018). Outmigration results in abandoned land (Badia et al. 2019), food security decline, decreased biodiversity, loss of multiple services from multifunctional land use, and a breakdown in social structure and cultural practices (Perpiña Castillo et al. 2020). Combating rural depopulation was the highest ranked motivation for initiative participation, with cultural heritage and increasing landscape beauty also among the most important motivations for land managers. Similar results have been found for integrated landscape initiatives in Europe (García-Martín et al. 2016).

Profitability is a major driver for stewardship of agroforestry landscapes and its lack is one of the main drivers of abandonment (Wolpert et al. 2020). “Increased income” through land management was important for many respondents. Most are only part time land managers—presumably small scale farming does not provide enough money to support livelihoods, and better incomes are sought in urban areas. Reversing this trend is needed to regain thriving, multifunctional agroforestry landscapes that offer livelihoods and well-being for people while preserving cultural landscapes (Howkins 2003). Some of the land managers in our study noted that their recently planted and carefully husbanded fruit trees were not even yielding yet, which shows commitment to the future. Eight percent of respondents reported “increase income” as a very weak motivation, finding it “very weak motivation” more often than any other motivation option. This may reflect the findings of Oviedo et al. (2017) that farmers are (if they can afford) often motivated as much if not more by amenities like living in nature and having a desirable lifestyle than by profits.

The movement of neo-ruralism is is getting more and more attention since it is a widespread trend in Europe (Bender and Kanitscheider 2012; Dal Bello et al. 2021). Neo-rurals are characterised as farmers that moved to rural areas as a response to the Green revolution and critique of city life (Escribano and Mormont 2007), seeking to protect biodiversity and grow high quality local food (Orria and Luise 2007). Previous research has also highlighted how rural environments are attracting neo-rurals as new entrepreneurs for various reasons, especially in search of a better quality of life (Dal Bello et al. 2021; Dall Bello et al. 2022). This is in line with our findings that showed that “increasing biodiversity”, “growing their own food” and “improving personal wellbeing” as more important motivations for neo-rurals compared to rurals in managing their land. Rurals were more motivated than neo-rurals by “mitigating climate change” which could be due to their own experience with changing climatic conditions, including drought. The high motivation to mitigate climate change in both groups is surprising as farmers seem to have a very low awareness of climate change globally (Madhuri 2020; Saliman and Petersen-Rockney 2022). In the current context of rural land abandonment, the incorporation of neo-rural populations may provide new opportunitites both for revitalising rural economies (Renau 2018; Dal Bello et al. 2022), and for the conservation of cultural landscapes (Pérez and Gurría 2010). As our results indicate, neo-rurals may show stronger motivations linked with pro-environmental behaviour. They might bring in innovative practices and think more globally. This could fruitfully complement the local traditional knowledge and experience of rural people. Collaboration among these groups could provide hope for the revival of cultural landscapes.

### Policy recommendations

Land managers perceived the lack of political support, and legislation not adapted to current fire conditions, as very strong barriers, even greater than a lack of funding. In other European initiatives, lack of funding was by far the biggest barrier identified (García-Martín et al. 2016). The reason may be uncontrolled forest expansion fostered by national and regional regulations that do not allow grazing in former forest areas, as described previously. García-Martín et al. (2016) found that among different professional groups, land managers in particular often have to cope with narrow and inflexible policies ill-matched to local conditions.

All land managers agreed with policy to “create a special land management regime for areas with high fire risk.” This would help land managers to better assess wildfire risk in their area and identify areas where management is needed. It could also provide a basis for territorial planning processes (Marey-Perez et al. 2021). “Decreasing bureaucratic requirements” was strongly supported by respondents. This can be an important step in making active land management more attractive and providing straightforward funding opportunities. Over 90% of land managers agreed that CAP subsidies for fire mitigation services, like grazing, would improve the situation and that a legal basis to allow grazing in forests is needed. Managing forests to decrease biomass reduces wildfire risk and increases efficiency of water use (Varela et al. 2020).

## Conclusion

The increase of megafires in the Mediterranean region requires new approaches for wildfire mitigation. The use of community-based agroforestry as a complement to top-down firefighting strategies is increasingly discussed. In our study of an integrated landscape initiative we found highy motivated land managers that perceived manifold beneficial personal and regional outcomes from such action. Our study offers the following key lessons:

- Integrated landscape initiatives not only help resolve land use conflicts, but may be extended to also support collaborative efforts to mitigate wildfires.
- Different land managers (livestock farmers, foresters, tree crop farmers, arable farmers) show high levels of agreement in their motivations for participating in integrated landscape management and in their perceptions of positive personal and regional outcomes from such an initiative.
- Wildfire mitigation through community-based agroforestry can also serve as leverage point for financing rural revival and provision of multiple ecosystem services.
- Neo-rurals and rurals differ in some of their perceptions and motivations. These might complement each other in efforts to revive landscapes that are being abandoned.
- Policy should support land management that reduces wildfire risk by adapting legislation and funding schemes.

## Supplementary Information

Below is the link to the electronic supplementary material.

Supplementary file1 (DOCX 69 KB)

## Data Availability

Anonymized raw data are archived on the Zenodo repository, 10.5281/zenodo.7157514
