# Supplementary material for: Collaborative agroforestry to mitigate wildfires in Extremadura, Spain: land manager motivations and perceptions of outcomes, benefits, and policy needs
Source: Agrofor Syst. 2022 Oct 10;96(8):1135–49. doi: 10.1007/s10457-022-00771-6 (PMC9550154; doi:10.1007/s10457-022-00771-6)
Supplement: Supplementary file 1 — Supplementary file1 (DOCX 69 KB) [file 10457_2022_771_MOESM1_ESM.docx]

SUPPLEMENTARY MATERIAL 1: SURVEY

Date .......................... Interviewee ID ..................... Name of Interviewer………………………………………

Interviewee Name ………………………………………………………………………………………………………………………...

We are researchers interested in the MOSAICO project, working with Fernando Pulido (University of Extremadura) and collaborating with the University of Kassel in Germany. We are interested in learning about the achievements of MOSAICO and would like to study this initiative as a model for future forest fire management and land use. Therefore, we would like to examine the impact that MOSAICO has on both the social and ecological sides. We are very interested in hearing your views. To answer our questions no knowledge is needed and there are no "right answers", we are only interested in your opinion. Moreover, the survey is totally anonymous.

Would you like to participate?
  Yes, I am interested in participating             No, I am not interested

May we take some pictures?  yes  no

**Section 1 – Land managers**

1.1 Where did you spend your childhood (until 10 years age approximately)?

on a farm  rural area/town < 2000 inhabitants
 rural area/town >2000 inhabitants  in a farmhouse  in a city

1.2 Which age range do you belong to?

18-35  36-50  51-65  >65

1.3 For how long have you been part of Mosaico? ………. Years

1.4 Do you perceive yourself as a neo-rural? (Someone who lived for a significant time in a city and has returned to a rural area to work and life there)
 yes  no

1.5 Is land management a tradition in your family?
 yes  no

1.6 Which of the following statements apply to your farming activities?
 You are the only person  You work with other family members
 It is a cooperation among 2-5 different workers
 It is a cooperation among >5 different workers

1.7 Does rural and neo-rural people work together on your farm?
 yes  no

1.8 How much of your total family income comes from your farming activities (excluding subsidies)?
 0-25%  26-50%  51-75%  76-100%

1.9 Earlier you mentioned the different activities on your farm. How would you say the profitability of each of them is (from very negative, negative, 0 neutral, positive, very positive)? (go back to question 1.3 to identify the activities chosen)

| Profitability of: | Very negativ  (-2) | Negative (-1) | Neutral (0) | Positiv (+1) | Very positive (+2) | Don´t know/no answer |
| --- | --- | --- | --- | --- | --- | --- |
|  | ☐ | ☐ | ☐ | ☐ | ☐ | ☐ |
|  | ☐ | ☐ | ☐ | ☐ | ☐ | ☐ |
|  | ☐ | ☐ | ☐ | ☐ | ☐ | ☐ |
|  | ☐ | ☐ | ☐ | ☐ | ☐ | ☐ |
|  | ☐ | ☐ | ☐ | ☐ | ☐ | ☐ |
|  | ☐ | ☐ | ☐ | ☐ | ☐ | ☐ |

**Section 2 – Land manager activities**

*("your activities" always refers to your project which is part of MOSAICO)*

2.1 What type of activities do you have within MOSAICO? Please chose only one option!

livestock  forestry for wood  forestry for biomass  agroforestry  cultivation

2.2 How long have you been managing your land?

< 2 years  2-5 years  6-10 years  11-30 years  >30 years

2.3 What activities/lines of business are included in your farm? (please, mark as many options as apply)

agroforestry  resin tapping forestry for wood
 forestry for biomass  grazing animals  dairy farming
 bee-keeping  poultry  vegetables
 cereals  herbs  chestnut trees
 cherry trees  olive trees  other fruit trees
 vineyards  wood fuel  aromatic plants
 others, namely……………………………………

2.4 How would you define yourself as a land manager?
....................................................................................................................................

4.5 How much time do you spend on the following activities on your farm?

|  |  | Of very low importance  (1) | Of low importance  (2) | Of medium importance  (3) | Of high importance  (4) | Of very high importance  (5) | I don´t know |
| --- | --- | --- | --- | --- | --- | --- | --- |
| a) | Crop processing (cheese, oil ...) |  |  |  |  |  |  |
| b) | Selling to consumers |  |  |  |  |  |  |
| c) | Increase of areas with agroforestry/forestry |  |  |  |  |  |  |
| d) | Planting fruit trees |  |  |  |  |  |  |
| e) | Training/education for you and the farmers on your farm |  |  |  |  |  |  |
| f) | Education/training and consultancy to other land managers |  |  |  |  |  |  |

**Section 3 – Aims/Motivation**

3.1 How important to you are the following reasons for engaging in land management and the initiative?

|  |  | Of very low importance  (1) | Of low importance  (2) | Of medium importance  (3) | Of high importance  (4) | Of very high importance  (5) | Don´t know/ no answer |
| --- | --- | --- | --- | --- | --- | --- | --- |
| a) | Knowledge sharing with other farmers |  |  |  |  |  |  |
| b) | Combating wildfires |  |  |  |  |  |  |
| c) | Belonging to a group of like-minded people |  |  |  |  |  |  |
| d) | Increasing income |  |  |  |  |  |  |
| e) | Increasing biodiversity |  |  |  |  |  |  |
| f) | Mitigating climate change |  |  |  |  |  |  |
| g) | Increasing soil fertility |  |  |  |  |  |  |
| h) | Growing own food |  |  |  |  |  |  |
| i) | Improving personal well-being |  |  |  |  |  |  |
| j) | Improving local livelihoods |  |  |  |  |  |  |
| k) | Preserving landscape beauty |  |  |  |  |  |  |
| l) | Preserving cultural heritage |  |  |  |  |  |  |
| m) | Combating depopulation |  |  |  |  |  |  |
| n) | Do something meaningful |  |  |  |  |  |  |
| o) | Others, namely ……….. |  |  |  |  |  |  |

**Section 4 - Outcomes/Performance**

4.1 How have the activities of your farm changed since you have been part of MOSAICO (referring to changes in processing, change from cultivation to agroforestry, change in techniques such as stopping to plough, or starting to sell on local markets, etc.)?
…………………………………………………………………………………………………………………………………………………………….

………………………………………………………………………………………………………………………………………………………….…

4.2 Could you tell us what were your expectations of MOSAICO before you joined?
…………………………………………………………………………………………………………………………………………………………….

………………………………………………………………………………………………………………………………………………………….……………………………………………………………………………………………………………………………………………………………….

4.3 How do you agree with the following statements about the effects of MOSAICO in the Gata-Hurdes area? MOSAICO has…

| **Statements** | I fully disagree  (1) | I mainly disagree  (2) | Neither/ nor  (3) | I mainly agree  (4) | I fully agree  (5) | I don´t know |
| --- | --- | --- | --- | --- | --- | --- |
| a) … helped to increase biodiversity |  |  |  |  |  |  |
| b) … helped to counteract abandonment |  |  |  |  |  |  |
| c) … increased the use of sustainable land-management practices |  |  |  |  |  |  |
| d) … helped to increase the local ecological knowledge |  |  |  |  |  |  |
| e) … helped combatting wild fires |  |  |  |  |  |  |
| f) … helped to improve the economy of Gata-Hurdes |  |  |  |  |  |  |
| g) … improved the wellbeing of local people |  |  |  |  |  |  |

4.4 How much do you agree or disagree with the statements below about the direct impacts of Mosaico on you and on your farm? Mosaico has…

| **Statements** | I fully disagree  (1) | I mainly disagree  (2) | Neither/ nor  (3) | I mainly agree  (4) | I fully agree  (5) | I don´t know |
| --- | --- | --- | --- | --- | --- | --- |
| a) … improved your personal wellbeing |  |  |  |  |  |  |
| b)… made you more enthusiastic about traditional landscape management |  |  |  |  |  |  |
| c) … put you in closer contact to other land managers to exchange knowledge and material |  |  |  |  |  |  |
| d) …helped you to develop a closer relationship to your customers/consumers |  |  |  |  |  |  |
| e) … fostered learning among experienced land managers and neo-rurals |  |  |  |  |  |  |
| f) … made you increase crop diversity |  |  |  |  |  |  |
| g) … made you increase the numbers of products you sell |  |  |  |  |  |  |
| h) … helped you to increase the number of customers |  |  |  |  |  |  |
| i) … helped to improve the profitability of our activities |  |  |  |  |  |  |
| j) … increased collaboration between you and other land managers |  |  |  |  |  |  |
| k) … helped you overcome administrative barriers |  |  |  |  |  |  |
| l) … fulfilled the expectations you had before you started to be part of MOSAICO |  |  |  |  |  |  |
| m) … helped improve technical performance and management skills |  |  |  |  |  |  |

4.5 What are the three most important things that other initiatives can learn from Mosaico? Please explain in a short sentence, with 1 being the most important.
1. ………………………………………………………………………………………………………………………………………………………..
2. ………………………………………………………………………………………………………………………………………………………..
3. …………………………………………………………………………………………………………………………………………………………

**Section 5 – Barriers to management success**

5.1 To what extent have the following factors negatively affected the success of your activities?

|  | **Statements** | Very little negatively affected  (1) | Little negatively affected  (2) | Neither too much nor too little negatively affected (3) | Quite negatively affected (4) | Heavily negatively affected (5) | I don’t know/no answer |
| --- | --- | --- | --- | --- | --- | --- | --- |
| a) | Lack of funding |  |  |  |  |  |  |
| b) | Lack of profitability |  |  |  |  |  |  |
| c) | Lack of land-management expert knowledge |  |  |  |  |  |  |
| d) | Lack of administrative and organisational know-how |  |  |  |  |  |  |
| e) | Plant and animal pests and diseases |  |  |  |  |  |  |
| f) | Challenging climate conditions |  |  |  |  |  |  |
| g) | Lack of political support |  |  |  |  |  |  |
| h) | Lack of adapted legislation |  |  |  |  |  |  |
| i) | Other, namely |  |  |  |  |  |  |

5.2 How do you think the following measures would improve the performance/operation of MOSAICO?

|  | Would improve very little (1) | Would improve little (2) | Neither a little nor a lot (3) | Would improve a lot (4) | Would improve a lot (5) | Don´t know/no answer |
| --- | --- | --- | --- | --- | --- | --- |
| a) Simplify the administrative process so that enterprises can be considered "productive firewalls" | ☐ | ☐ | ☐ | ☐ | ☐ | ☐ |
| b) To create a special land management regime for Areas with High Fire Risk |  |  |  |  |  |  |
| c) To amend the National Forestry Law to allow strategic agricultural uses within forest areas |  |  |  |  |  |  |
| d) Make changes to the CAP to subsidise the fire prevention service provided by farmers |  |  |  |  |  |  |
| e) Other: |  |  |  |  |  |  |

**Section 6 – Success factors for initiatives goals**

6.1 How important are the following characteristics for the success of MOSAICO?

| **Feature** | Very unimportant  (1) | Of little importance  (2) | Neutral  (3) | Quite important  (4) | Very important  (5) | Don´t know/ no answer |
| --- | --- | --- | --- | --- | --- | --- |
| a) To have a common goal |  |  |  |  |  |  |
| b) Shared experiences |  |  |  |  |  |  |
| c) A diversity of viewpoints and skills |  |  |  |  |  |  |
| d) Active participation of members |  |  |  |  |  |  |
| e) Conflict facilitation |  |  |  |  |  |  |
| f) Fighting a common and immediate threat (like fire risk) |  |  |  |  |  |  |
| g) Knowledge sharing |  |  |  |  |  |  |
| h) Cooperation between different stakeholders and sectors |  |  |  |  |  |  |
| i) Other: |  |  |  |  |  |  |

**Section 7 - Wildfires**

7.1 How helpful are the following measures for mitigating wild-fires in your opinion?

|  | **Measure** | Very unhelpful  (1) | Unhelpful  (2) | Neither unhelpful nor helpful (3) | Helpful  (4) | Very helpful  (5) | I don´t know |
| --- | --- | --- | --- | --- | --- | --- | --- |
| a) | Implement more/new regulations |  |  |  |  |  |  |
| b) | Strengthening conventional fire prevention measures |  |  |  |  |  |  |
| c) | Increase resources for conventional fire management (e.g. more helicopters) |  |  |  |  |  |  |
| d) | Promoting forestry |  |  |  |  |  |  |
| e) | Promote animal grazing as a productive firewall |  |  |  |  |  |  |
| f) | Promoting cultivation as a productive firewall |  |  |  |  |  |  |
| g) | Promote tree crops/agroforestry as productive firebreaks |  |  |  |  |  |  |

7.2 To what extent do you agree or disagree with the following statements regarding wildfires?

| **Statements** | Fully disagree  (1) | Rather disagree  (2) | Neither/ nor (3) | Rather agree  (4) | Fully agree  (5) | I don´t know |
| --- | --- | --- | --- | --- | --- | --- |
| a) Wildfires are the main reason for people to get involved in Mosaico |  |  |  |  |  |  |
| b) You were strongly affected by wildfires |  |  |  |  |  |  |
| c) Wild-fires caused a physical damage to your farm |  |  |  |  |  |  |
| d) Wildfires caused psychological distress for a person that belongs to your farm |  |  |  |  |  |  |
| e) Fires were the main reason for joining MOSAICO |  |  |  |  |  |  |

**To fill by the interviewer:**

- Place of the survey:................................................................................................................................

- Attitude of the respondent: good/ indifferent/ unwilling

- Understanding of the questionnaire: high/medium/low

- Sex of respondent: male/female

SUPPLEMENTARY MATERIAL 2: Statistical analysis of rural versus neo-rural land managers motivations (table 1), outcomes (table 2), wildfire measures (table 3) and barriers (table 4).

Table 1 Motivations of rural and neo-rural land managers to work in land
management and join the initiative. Information include: Mann Whitney U, as
well as the p-value. Statistically significant differences are marked in bold (p<0.05).

| **Motivations** | **Mann Whitney U** | **p-value** |
| --- | --- | --- |
| knowledge sharing | 471.0 | 0.86 |
| combating wildfires | 444.5 | 0.17 |
| like-minded people | 477.5 | 0.61 |
| increasing income | 548.0 | 0.21 |
| increasing biodiversity | 387.5 | **0.01** |
| mitigating climate change | 409.5 | **0.02** |
| increasing soil fertility | 455.0 | 0.13 |
| growing own food | 273.5 | **0.003** |
| improving personal well-being | 323.5 | **0.01** |
| improving local livelihoods | 447.0 | 0.75 |
| preserving landscape beauty | 420.5 | 0.43 |
| preserving cultural heritage | 499.5 | 0.56 |
| combating depopulation | 522.5 | 0.20 |
| do something meaningful | 472.5 | 0.36 |

Table 2 Barriers to success of rural and neo-rural land managers to
 work on their farm. Information include:) Mann Whitney U, as well as
 the p-value. Statistically significant differences are marked in bold (p<0.05).

| **Barriers to success** | **Mann Whitney U** | **p-value** |
| --- | --- | --- |
| lack of funding | 480.5 | 0.86 |
| lack of profitability | 518.0 | 0.47 |
| lack of experts | 419.0 | 0.47 |
| lack of administrative know-how | 482.0 | 0.85 |
| pest and diseases | 320.0 | **0.03** |
| challenging climate conditions | 402.0 | 0.33 |
| lack of politcal support | 473.5 | 0.95 |
| lack of adapted legislation | 409.5 | 0.35 |

Table 3 Personal and regional outcomes of rural and neo-rural land managers.
 Information include: Mann Witney U, as well as the p-value. There are no
statistically significant differences (p<0.05).

| **Outcomes (regional and personal)** | **Mann Whitney U** | **p-value** | |  |  |  |  |
| --- | --- | --- | --- | --- | --- | --- | --- |
| increase biodiversity | 437.0 | 0.68 | |  |  |  |  |
| counteract abandonment | 402.5 | 0.16 | |  |  |  |  |
| increase sustainable land management | 491.5 | 0.72 | |  |  |  |  |
| increase local ecological knowledge | 488.0 | 0.96 | |  |  |  |  |
| combating wild-fires | 447.5 | 0.76 | |  |  |  |  |
| improved regional economy | 503.5 | 0.46 | |  |  |  |  |
| improved well-being of locals | 456.5 | 0.52 | |  |  |  |  |
| improved personal wellbeing | 462.5 | 0.94 | |  |  |  |  |
| more enthusiastic about traditional land management | 507.0 | 0.57 | |  |  |  |  |
| exchange knowledge | 437.5 | 0.66 | |  |  |  |  |
| closer contact to consumers | 558.5 | 0.19 | |  |  |  |  |
| fostered learning among rurals and non-rurals | 469.0 | 0.99 | |  |  |  |  |
| increase crop diversity | 494.5 | 0.70 | |  |  |  |  |
| increase products sold | 497.0 | 0.68 | |  |  |  |  |
| increase customers | 511.0 | 0.53 | |  |  |  |  |
| increase profitability | 514.0 | 0.50 | |  |  |  |  |
| increase collaboration among farmers | 420.0 | 0.48 | |  |  |  |  |
| helped overcome administrative barriers | 457.5 | 0.88 | |  |  |  |  |
| fullfilled expectations | 435.0 | 0.63 | |  |  |  |  |
| increased management skills | 378.0 | 0.18 | |  |  |  |  |
|  |  | |  | |  |  |  |

Table 4 Helpfulness of wildfire related practices of rural and neo-rural land
managers. Information include: Mann Witney U, as well as the p-value.
 Statistically significant differences are marked in bold (p<0.05).

| **Wildfire related measures** | **Mann Whitney U** | **p-value** |
| --- | --- | --- |
| more regulations | 512.50 | 0.51 |
| strengthening prevention | 424.00 | 0.41 |
| increase resources for conventional measures | 446.50 | 0.74 |
| promote forestry | 385.50 | 0.13 |
| promote grazing | 488.00 | **< 0.0001** |
| promote cultivation | 483.00 | **< 0.0001** |
| promote agroforestry | 452.00 | **< 0.0001** |
| wildfires as reason joining Mosaico | 496.50 | 0.70 |
| affected by wildfires | 525.50 | 0.41 |
| caused physical damage to initiative | 522.00 | 0.43 |
